# Pathogenicity of new *BEST1* variants identified in Italian patients with best vitelliform macular dystrophy assessed by computational structural biology

**DOI:** 10.1186/s12967-019-2080-3

**Published:** 2019-10-01

**Authors:** Vladimir Frecer, Giancarlo Iarossi, Anna Paola Salvetti, Paolo Enrico Maltese, Giulia Delledonne, Marta Oldani, Giovanni Staurenghi, Benedetto Falsini, Angelo Maria Minnella, Lucia Ziccardi, Adriano Magli, Leonardo Colombo, Fabiana D’Esposito, Jan Miertus, Francesco Viola, Marcella Attanasio, Emilia Maggio, Matteo Bertelli

**Affiliations:** 1 Department of Physical Chemistry of Drugs, Faculty of Pharmacy, Comenius University in Bratislava, Bratislava, Slovakia; 2 Department of Ophthalmology, Bambino Gesù IRCCS Children’s Hospital, Rome, Italy; 3 Department of Biomedical and Clinical Sciences “Luigi Sacco”, Sacco Hospital, University of Milan, Milan, Italy; 4 MAGI’S Lab S.R.L., Via Delle Maioliche 57/D, 38068 Rovereto, TN Italy; 5 Institute of Ophthalmology, Visual Electrophysiology Service, Fondazione Policlinico Gemelli/UniversitàCattolica del S. Cuore, Rome, Italy; 6 Neurophthalmology and Neurophysiology Unit, GB Bietti Foundation-IRCCS, Rome, Italy; 7 Department of Ophthalmology, Orthoptic and Pediatric Ophthalmology, University of Salerno, Salerno, Italy; 8 Department of Ophthalmology, San Paolo Hospital, University of Milan, Milan, Italy; 9 Imperial College Ophthalmic Research Unit, Western Eye Hospital, Imperial College Healthcare NHS Trust, London, UK; 10 Eye Clinic, Department of Neurosciences, Reproductive Sciences and Dentistry, University of Naples Federico II, Naples, Italy; 11 MAGI Euregio, Bolzano, Italy; 12 Genius n.o., Trnava, Slovakia; 13 Department of Ophthalmology, Fondazione IRCCS Cà Granda, Clinica Regina Elena, Milan, Italy; 14 IRCCS-Ospedale Sacro Cuore Don Calabria, Negrar, VR Italy

**Keywords:** Best vitelliform macular dystrophy, Best disease, Best-corrected visual acuity, Computational structural biology

## Abstract

**Background:**

Best vitelliform macular dystrophy (BVMD) is an autosomal dominant macular degeneration. The typical central yellowish yolk-like lesion usually appears in childhood and gradually worsens. Most cases are caused by variants in the *BEST1* gene which encodes bestrophin-1, an integral membrane protein found primarily in the retinal pigment epithelium.

**Methods:**

Here we describe the spectrum of *BEST1* variants identified in a cohort of 57 Italian patients analyzed by Sanger sequencing. In 13 cases, the study also included segregation analysis in affected and unaffected relatives. We used molecular mechanics to calculate two quantitative parameters related to calcium-activated chloride channel (CaCC composed of 5 BEST1 subunits) stability and calcium-dependent activation and related them to the potential pathogenicity of individual missense variants detected in the probands.

**Results:**

Thirty-six out of 57 probands (63% positivity) and 16 out of 18 relatives proved positive to genetic testing. Family study confirmed the variable penetrance and expressivity of the disease. Six of the 27 genetic variants discovered were novel: p.(Val9Gly), p.(Ser108Arg), p.(Asn179Asp), p.(Trp182Arg), p.(Glu292Gln) and p.(Asn296Lys). All *BEST1* variants were assessed in silico for potential pathogenicity. Our computational structural biology approach based on 3D model structure of the CaCC showed that individual amino acid replacements may affect channel shape, stability, activation, gating, selectivity and throughput, and possibly also other features, depending on where the individual mutated amino acid residues are located in the tertiary structure of BEST1. Statistically significant correlations between mean logMAR best-corrected visual acuity (BCVA), age and modulus of computed BEST1 dimerization energies, which reflect variations in the in CaCC stability due to amino acid changes, permitted us to assess the pathogenicity of individual *BEST1* variants.

**Conclusions:**

Using this computational approach, we designed a method for estimating BCVA progression in patients with *BEST1* variants.

## Background

Best vitelliform macular dystrophy (BVMD) (OMIM #153700), also known as Best’s disease, is an autosomal dominant slowly progressive form of retinal macular degeneration. The typical central yellowish yolk-like lesion due to accumulation of lipofuscin in the retinal pigment epithelium (RPE) usually appears in childhood. The lesion gradually worsens [1] causing progressive macular atrophy or fibrosis and subsequent loss of visual acuity [2].

Five stages of the disease have been described: in the first or pre-vitelliform stage, usually discovered incidentally, subtle RPE alterations of the macula cause no symptoms. The second or vitelliform stage is characterized by a well-defined 0.5 to 2 mm diameter “egg-yolk” lesion in the macula, and patients may experience symptoms such as metamorphopsia, blurred vision and a decrease in visual acuity. In stage 3, this deposit is partially reabsorbed and deposited in a layer in the macula, known as “pseudohypopyon”. In the fourth or vitelliruptive stage, there is partial reabsorption of the material (scrambled-egg lesion) and macular atrophy. Macular fibrosis develops in stage 5 [3].

The vast majority of cases are caused by a variant in the *BEST1* gene (also known as *VMD2*) which encodes bestrophin-1 (BEST1) [4].

**Fig. 1:**
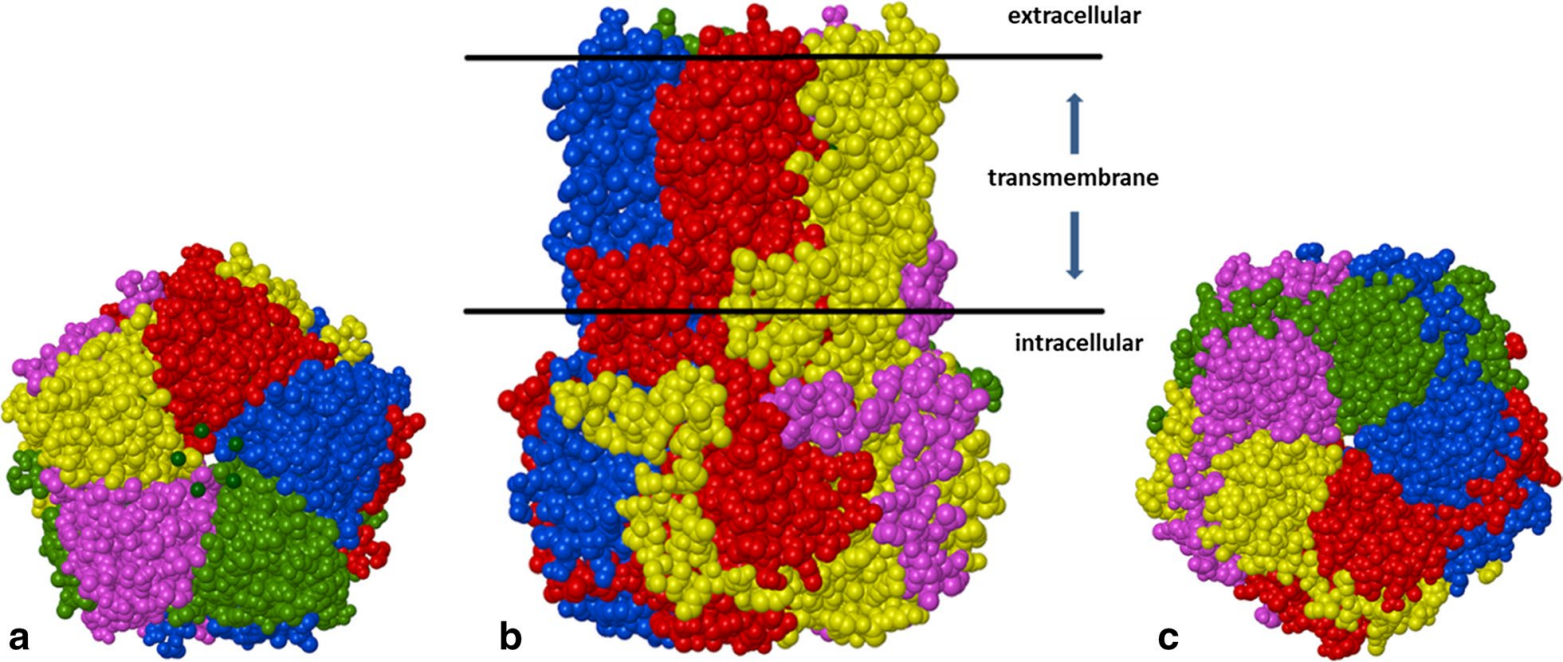
X-ray structure of bestrophin-1 from *Gallus gallus* [9, 10] forming a calcium-activated chloride transmembrane channel (CaCC) composed of five identical subunits and containing a single long pore along the axis of symmetry. **a** Top view (extracellular side), **b** side view, **c** bottom view (cytosol side, channel aperture). The CaCC is shown in Corey–Pauling–Koltun (CPK) representation, dark green spheres—chloride ions (A), orange spheres—potassium ions (not visible), blue spheres—calcium ions (not visible)

Bestrophin-1 protein is expressed in the basolateral membrane of the RPE [4]. It is essential for normal eye development during embryogenesis and for retinal homeostasis throughout life [5]. Bestrophin-1 belongs to the family of calcium-activated chloride channels (CaCCs) that regulate the flow of chloride and other monovalent anions across cell membranes in response to intracellular Ca^2+^ levels [6–8]. These channels occur in various types of cell. The mechanisms of CaCC anion selectivity, calcium-dependent gating and the bestrophin-1 domains involved in channel regulation are not fully understood. The X-ray structure of BEST1 from *Gallus gallus,* which shares 74% sequence identity with human BEST1, showed that five bestrophin-1 molecules compose a CaCC, which contains a single long pore along a five-fold symmetry axis (Fig. 1) [9, 10]. The pore extends from the extracellular side across the cell membrane and through the cytosol. Its variable diameter defines two narrow hydrophobic regions: the neck and the aperture, which are thought to be related to channel gating and anion selectivity [9, 10]. The X-ray structure of BEST1 CaCC also shows five calcium-binding sites in the cytosolic portions of the subunits (Ca^2+^ clasps) and three binding sites for Cl^−^ for each subunit in the channel pore [9, 10].

**Fig. 2:**
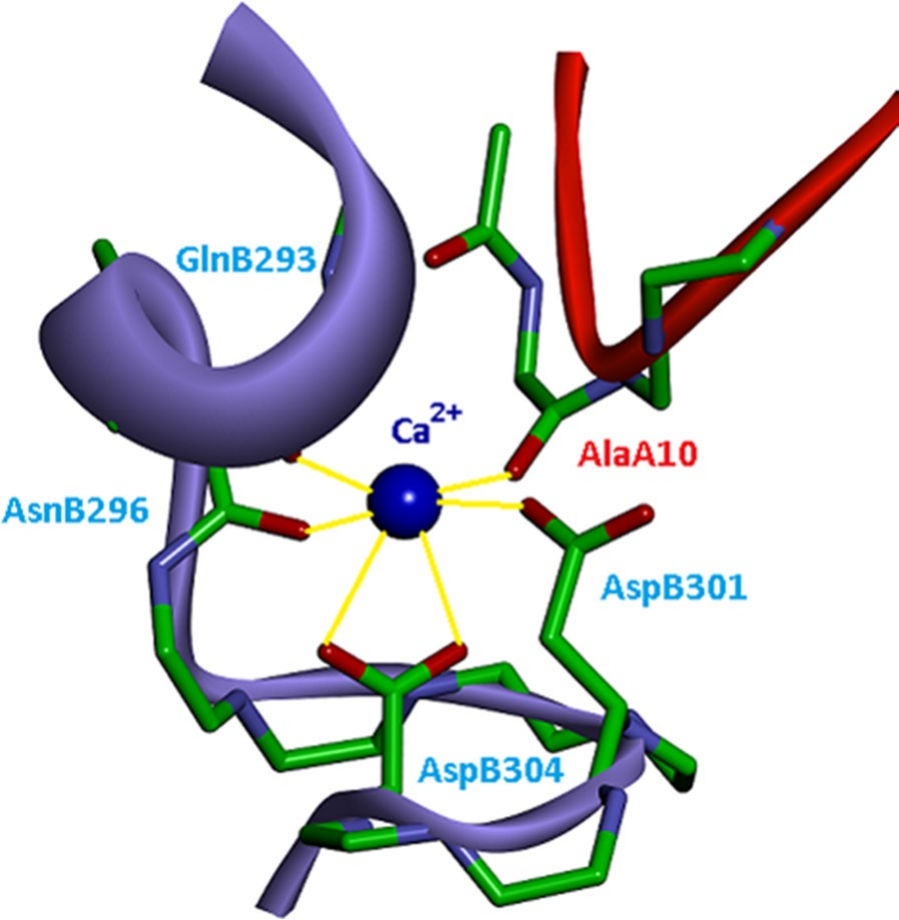
Crystal structure of the “Ca^2+^ clasp” of BEST1 from *Gallus gallus* [9, 10]. The calcium binding site is formed by two bestrophin-1 subunits represented by the red (subunit A) and blue ribbons (subunit B) and is involved in the CaCC activation. Ca^2+^ is shown as a dark blue sphere. Yellow lines indicate calcium ion coordination by residues side chains and backbone carbonyl groups of both subunits. A water molecule coordinating the calcium ion in the crystal structure is not shown

The N-terminal region of bestrophin-1 (residues 1–390 in human BEST1) is highly conserved in eukaryotic CaCCs and has been shown sufficient for CaCC activity and Cl^−^ ion transport [11]. The C-terminal cytosolic portion of the protein (amino acids 391–585) is predicted to be unstructured with an as yet undefined role in channel regulation [10]. A water molecule, acidic residues Asp301, Asp304 and backbone carbonyl groups of two neighboring bestrophin-1 subunits cooperate in calcium ion coordination in the Ca^2+^ clasps [9, 10], Fig. 2.

Most of the approximatively 200 known disease-causing variants in *BEST1* associated with retinal degenerative disorders have been reported to induce CaCC aberrations [5, 12–15]. Alterations in BEST1 CaCC functions lead to a diminished electrooculogram (EOG) light peak to dark trough ratio typical of BVMD [16]. BVMD is a heterogeneous pleomorphic disease underling different phenotypes and with alternative modes of inheritance. Although most cases show autosomal dominant inheritance, recessive inheritance has also been described [7, 9, 10]. Patients with recessive inheritance may have the classic features of Best disease [17], including a central vitelliform lesion, or may have extramacular punctate flecks without any notable central lesion [18]. Variants in *BEST1* are also responsible for other clinically distinct human diseases: autosomal dominant vitreoretinochoroidopathy (ADVIRC) (OMIM #193220), autosomal recessive bestrophinopathy (ARB) (OMIM #611809) and retinitis pigmentosa (RP) (OMIM #613194) [19].

Here we report the results of the characterization of *BEST1* variants in an Italian population consisting of 57 probands and 13 families, thus contributing to the molecular epidemiology of Best disease in our country. Six of the 27 *BEST1* variants detected are novel.

All variants were assessed by computational structural biology. The conclusions of computational modelling led to the formulation of simple approximate quantitative structure-pathogenicity relationships (QSPR) of the *BEST1* variants and a tool for predicting visual acuity progression in individuals with inherited *BEST1* variants.

## Methods

### Genetic testing

Fifty-seven probands with clinical features suggesting Best’s disease were examined in different eye clinics and hospitals. Most of the patients underwent complete ophthalmic examination, including logMAR best-corrected visual acuity (BCVA), anterior and posterior segment examination and retinal imaging. Imaging included fundus photography (Zeiss, Visucam, Oberkochen, Germany), spectral domain optical coherence tomography (SD-OCT, Spectralis HRA + OCT, Heidelberg Engineering, Heidelberg, Germany), infra-red (IR) imaging and blue fundus autofluorescence (B-FAF) (Spectralis HRA and HRA II, Heidelberg Engineering). Some patients also underwent electrophysiological testing including electro-oculogram and multifocal electroretinogram [EOG, mfERG, VERIS Clinic 4.9, Electro-Diagnostic Imaging, San Mateo, CA and Retimax instrument (CSO, Firenze, Italy)] recordings and ultra-structural morphology evaluation by adaptive optics (AO, rtx1, Imagine Eyes, Orsay, France) retinal imaging. A small number of patients were only addressed to our laboratories for genetic testing from different institutions, therefore with partial clinical data.

A genetic test was performed to confirm the diagnosis of Best disease; whenever possible, ophthalmic examination and genetic analysis were extended to members of the proband’s family.

A total of 57 blood samples from patients and 18 from family members were received and analyzed by MAGI Laboratory (MAGI’s Lab, Rovereto, Italy). DNA was extracted by commercial kit (Blood DNA Kit E.Z.N.A.; Omega Bio-Tek Inc., Norcross, GA, USA). Extracted DNA underwent polymerase chain reaction (PCR) to amplify all coding regions (exons 2–11) and the intron/exon junctions of *BEST1* (NM_004183.3). The products were purified and sequenced with a Beckman Coulter CEQ 8000 sequencer (Beckmann Coulter, Milan, Italy). All laboratory protocols are available upon request.

The electropherograms of all amplified fragments were analyzed with ChromasPro 1.5 software (Technelysium Pty Ltd, Australia; http://technelysium.com.au) and then compared with the reference sequences using Basic Local Alignment Search Tool 2 (BLAST2) sequences (http://blast.ncbi.nlm.nih.gov).

For the identification of variants, the Retina International database (http://www.retina-international.org), the Human Gene Mutation Database Professional version 2017.2 (https://portal.biobase-international.com/hgmd/pro/), the Exome Variant Server (http://evs.gs.washington.edu/EVS/) and dbSNP (https://www.ncbi.nlm.nih.gov/snp/) were also consulted.

New variants were analyzed for their putative pathogenicity using three on-line softwares: Polyphen 2 (Polymorphism Phenotyping v2; http://genetics.bwh.harvard.edu/pph2), SIFT (Sorting Intolerant From Tolerant; http://sift.bii.a-star.edu.sg/) and Mutation Taster (http://mutationtaster.org/). Pedigrees were designed according to Bennett et al. [20] using HaploPainter software (http://haplopainter.sourceforge.net/). New variants were classified according to the American College of Medical Genetics and Genomics standards and guidelines [21].

### Molecular modelling

A refined 3D template model of human CaCC was prepared from the X-ray structure of chicken BEST1 (PDB entries 4RDQ and 5T5 N, 409 residues, resolution 3.1 Å) [9, 10] by protein homology modelling and molecular geometry optimization. Amino acid sequences of the N-terminal domain of *Homo sapiens* bestrophin-1 (UniProtKB O76090, residues 1-366) and of bestrophin-1 from *Gallus gallus* were aligned by the EMBOSS Needle sequence alignment server (73.6% identity, 88.3% similarity, gaps 0.5%) (https://www.ebi.ac.uk/Tools/psa/emboss_needle/). The 3D homology model of human bestrophin-1 channel subunit A was built by comparative modelling from the chicken CaCC template structure with the help of the Prime protein structure prediction approach [22] using standard parameters (Schrödinger, LLC, New York, 2014). The loops of the human bestrophin-1 model were perfected using the *refine loops* tool of Prime. The homology model was inspected with help of Prime to ensure that there was no violation of protein stereochemistry. The human BEST1 model contained one Ca^2+^ cation, two K^+^, three Cl^−^ and one water molecule coordinating the calcium ion. After building the 3D model of BEST1 protein, five protein subunits were superimposed on the chicken CaCC template structure using the Prime structural alignment module (Schrödinger, LLC, New York, 2014) to obtain the quaternary structure of the human chloride channel. A dimer formed by two BEST1 subunits was separated from the CaCC model and further refined to convergence by energy-minimization using molecular mechanics (MM). During minimization, the OPLS-2005 force field [23–25] and the generalized Born implicit solvation model (GB/SA) [26] were employed with MacroModel software (Schrödinger LLC, New York, NY, 2014). At first, the protein backbone atoms and all ions were constrained in their initial positions, while all other atoms were unrestrained. In the final minimization step, all atoms were set free to find their relaxed positions in the model structure of human bestrophin-1 subunits.

To study the effect of individual variants detected in proband*s*, residue replacement with optimal side chain rotamer selection were conducted in the BEST1 dimer using MacroModel, followed by extensive dimer and monomer structure minimization. The consequence of variants for the bestrophin-1 subunit binding to form the pentameric channel and of subunit binding capacity to Ca^2+^ ions needed for channel regulation were estimated with the help of relative dimerization and calcium binding energies (ΔΔE_dim_ and ΔΔE_Cabin_) computed with respect to the native BEST1 protein. The ΔΔE_dim_ energy estimates the extent of possible damage or change in CaCC formation, expressed as the change in binding energy between neighboring bestrophin-1 subunits A and B in CaCC, caused by a variant (var), as compared to the reference native BEST1 [27–29]:

1 $$\Delta \Delta {\text{E}}_{ \dim } = \Delta {\text{E}}_{ \dim } \left\{ {{\text{BEST}}1_{{{\text{var}},{\text{AB}}}} } \right\}_{\text{aq}} - \Delta {\text{E}}_{ \dim } \left\{ {{\text{BEST}}1_{{{\text{native}},{\text{AB}}}} } \right\}_{\text{aq}}$$

2 $$\Delta {\text{E}}_{ \dim } \left\{ {{\text{BEST}}1_{{{\text{var}},{\text{AB}}}} } \right\}_{\text{aq}} = {\text{E}}_{\text{tot}} \left\{ {{\text{BEST}}1_{{{\text{var}},{\text{AB}}}} } \right\}_{\text{aq}} - {\text{E}}_{\text{tot}} \left\{ {{\text{BEST}}1_{{{\text{var}},{\text{A}}}} } \right\}_{\text{aq}} - {\text{E}}_{\text{tot}} \left\{ {{\text{BEST}}1_{{{\text{var}},{\text{B}}}} } \right\}_{\text{aq}}$$

where E_tot_{Z}_aq_ is the total molecular mechanics (MM) energy of hydrated {}_aq_ protein Z (dimer AB or monomers A and B) computed by MacroModel (Schrödinger LLC, New York, NY, 2014). The relative Ca^2+^ binding energy (ΔΔE_Cabin_) reflects the extent of possible damage or change to channel regulation expressed as the altered ability of the BEST1 dimer AB to bind calcium ions due to a variant, with respect to the reference native BEST1:

3 $$\Delta \Delta {\text{E}}_{\text{Cabin}} = \Delta {\text{E}}_{\text{Cabin}} \left\{ {{\text{BEST}}1_{{{\text{var}},{\text{AB}},{\text{Ca}}}} } \right\}_{\text{aq}} - \Delta {\text{E}}_{\text{Cabin}} \left\{ {{\text{BEST}}1_{{{\text{native}},{\text{AB}},{\text{Ca}}}} } \right\}_{\text{aq}}$$

4 $$\Delta {\text{E}}_{\text{Cabin}} \left\{ {{\text{BEST}}1_{{{\text{var}},{\text{AB}},{\text{Ca}}}} } \right\}_{\text{aq}} = \frac{1}{2}\left[ {{\text{E}}_{\text{tot}} \left\{ {{\text{BEST}}1_{{{\text{var}},{\text{AB}},2{\text{Ca}}}} } \right\}_{\text{aq}} - {\text{E}}_{\text{tot}} \left\{ {{\text{BEST}}1_{{{\text{var}},{\text{AB}}}} } \right\}_{\text{aq}} - 2{\text{E}}_{\text{sol}} \left\{ {{\text{Ca}}^{2 + } } \right\}_{\text{aq}} } \right]$$

where E_tot_{Z}_aq_ and E_sol_{Ca^2+^}_aq_ are the total MM energy of the hydrated bestrophin-1 dimer with or without bound calcium ions, or the solvation energy of the Ca^2+^ ion.

QSPR of *BEST1* variants affecting the visual acuity of the probands were elaborated with help of the Cerius^2^ software package (Cerius^2^ Life Sciences, version 4.5, 2000. Accelrys, San Diego, CA, USA) [30, 31]. Clinically evaluated stages and visual acuities averaged over both eyes of a proband and averaged over probands carrying the same *BEST1* variant were correlated by linear regression with the computed subunit dimerization and calcium binding energies (ΔΔE_dim_, ΔΔE_Cabin_) as well as the modulus (absolute value) of dimerization energy, |ΔΔE_dim_|. Due to the lack of other functional parameters such as the Arden Index or Mf-ERG for all the patients other correlations were not possible. A set of 20 distinct *BEST1* variants in 27 probands, for whom BCVA data was available (Table 1), was entered in the QSPR model. Statistical techniques for validation of the regression (leave-one-out cross-validation) were used to identify outliers (data points modelled poorly by the regression equation). In the Cerius^2^, an outlier is defined as a data point with a residual more than twice the standard deviation of the residuals generated in the validation procedure.

**Table 1:** Demographic, genetic and clinical characteristics of probands

Proband ID	Gender	Age^a^ [years]	Exon/intron	Nucleotide change	Amino acid change	Allele state	dbSNP/ClinVar accession number	Variant classification^b^	MAF	New or known variants	Best’s disease stage^c^		BCVA^d^	
											LE	RE	LE	RE
P1	M	50	ex2	c.5C>T	p.(Thr2Ile)	HET	NA	*Likely pathogenic*	NA	Kinnick (2011)	5	5	0.1	0.4
P2	F	42	ex2	c.11C>T	p.(Thr4Ile)	HET	NA	*Likely pathogenic*	NA	Tian (2014)	2	2	0	0
P3*	F	13	ex2	c.11C>T	p.(Thr4Ile)	HET	NA	*Likely pathogenic*	NA	Kinnick (2011)	1	1	0.1	0.1
P4*	M	27	ex2	c.26T>G	p.(Val9Gly)	HET	SCV000599452	*Likely pathogenic*	NA	NEW	2	2	0	0.6
P5	F	34	ex2	c.44G>A	p.(Gly15Asp)	HET	NA	*Likely pathogenic*	NA	Querques (2009)	NA	NA	NA	NA
P6	M	22	ex2	c.44G>A	p.(Gly15Asp)	HET	NA	*Likely pathogenic*	NA	Querques (2009)	3	4	0	0.2
P7	F	13	ex2	c.44G>A	p.(Gly15Asp)	HET	NA	*Likely pathogenic*	NA	Querques (2009)	0	3	0	0.22
P8*	M	56	ex2	c.47C>T	p.(Ser16Phe)	HET	rs281865210	*Likely pathogenic*	NA	Marchant (2001)	2	2	0.1	0.7
P9	F	23	ex2	c.74G>A	p.(Arg25Gln)	HET	rs281865215	*Likely pathogenic*	A = 0.000008 (Topmed)	Marquardt (1998)	4	2	0.5	0
P10	F	47	ex1	c.80G>C	p.(Ser27Thr)	HET	NA	*Likely pathogenic*	NA	Bernardis (2016)	3	4	0.1	0.2
P11*	F	55	ex2	c.80G>C	p.(Ser27Thr)	HET	NA	*Likely pathogenic*	NA	Bernardis (2016)	2	2	NA	NA
P12	F	52	ex2	c.80G>C	p.(Ser27Thr)	HET	NA	*Likely pathogenic*	NA	Bernardis (2016)	3	3	0.7	1
P13*	M	10	ex2	c.86A>G	p.(Tyr29Cys)	HET	NA	*Likely pathogenic*	NA	Downs (2007)	4	4	0	0
P14	F	27	ex4	c.274C>T	p.(Arg92Cys)	HOM	rs281865224	Pathogenic	NA	Bakall (1999)	NA	NA	NA	NA
P15	M	42	ex4	c.278G>C	p.(Trp93Ser)	HET	NA	*Likely pathogenic*	NA	Tian (2017)	NA	NA	NA	NA
P16*	M	70	ex4	c.301C>A	p.(Pro101Thr)	HET	rs281865229	*Likely pathogenic*	NA	Lotery (2000)	4	3	0.5	0
P17	M	26	ex4	c.324C>G	p.(Ser108Arg)	HET	SCV000747137	*Vus*	NA	NEW	2	2	0.1	1
P18	M	46	ex5	c.535A>G	p.(Asn179Asp)	HET	SCV000599453	*Likely pathogenic*	NA	NEW	1	2	0.3	0.3
P19	F	13	ex5	c.544T>C	p.(Trp182Arg)	HET	SCV000747139	*Vus*	NA	NEW	4	4	NA	NA
P20*	M	19	ex5	c.598C>T	p.(Arg200*)	HET	rs121918286	Pathogenic	T = 0.00002/3 (ExAC)	Burgess (2008)	NA	NA	NA	NA
			ex7	c.728C>A	p.(Ala243Glu)	HET	NA	*Likely pathogenic*	NA	Fung (2014)	NA	NA	NA	NA
P21*	F	25	ex6	c.652C>T	p.(Arg218Cys)	HET	rs281865238	Pathogenic	NA	Johnson (2013)	2	2	0.1	0
P22	F	13	ex6	c.652C>T	p.(Arg218Cys)	HET	rs281865238	Pathogenic	NA	Johnson (2013)	0	3	0	0.17
P23	M	14	ex6	c.652C>T	p.(Arg218Cys)	HET	rs281865238	Pathogenic	NA	Johnson (2013)	2	2	0.4	0
P24	M	42	ex6	c.652C>A	p.(Arg218Ser)	HET	rs281865238	*Likely pathogenic*	NA	Bakall (1999)	3	3	0.5	0.4
P25	M	54	ex5	c.695T>A	p.(Ile232Asn)	HET	NA	*Likely pathogenic*	NA	Wabbels (2006)	3	3	0.6	0.8
P26	M	17	ex6	c.703G>T	p.(Val235Leu)	HET	NA	*Likely pathogenic*	NA	Marchant (2001)	3	3	0.1	0.1
P27*	F	51	ex7	c.727G>A	p.(Ala243Thr)	HET	rs137853905	Pathogenic	NA	Lotery (2000)	2	2	1	1
P28	M	49	ex7	c.728C>T	p.(Ala243Val)	HET	rs28940570	Pathogenic	T = 0.000008/1 (ExAC)	White (2000)	3	3	0.1	0
P29*	F	43	ex7	c.728C>T	p.(Ala243Val)	HET	rs28940570	Pathogenic	T = 0.000008/1 (ExAC)	White (2000)	3	2	0.2	0.2
P30*	F	50	ex7	c.728C>T	p.(Ala243Val)	HET	rs28940570	Pathogenic	T = 0.000008/1 (ExAC)	White (2000)	NA	NA	NA	NA
P31*	M	9	ex8	c.874G>C	p.(Glu292Gln)	HET	SCV000747140	*Likely pathogenic*	NA	NEW	2	2	1	1
P32	M	39	ex8	c.888C>G	p.(Asn296Lys)	HET	SCV000802932	*Pathogenic*	NA	NEW	3	3	0.1	0.3
P33	F	41	ex8	c.888C>A	p.(Asn296Lys)	HET	NA	*Pathogenic*	NA	Boon (2007)	2	2	0.3	0.3
P34	M	10	ex8	c.893T>G	p.(Phe298Cys)	HET	NA	*Likely pathogenic*	NA	Meunier (2011)	NA	NA	NA	NA
P35	M	53	ex8	c.893T>G	p.(Phe298Cys)	HET	NA	*Likely pathogenic*	NA	Meunier (2011)	NA	NA	NA	NA
P36*	M	21	ex8	c.903T>G	p.(Asp301Glu)	HET	rs281865261	Pathogenic	NA	Caldwell (1999)	NA	NA	0	0.6

*HET* heterozygous, *HOM* homozygous, rs# single nucleotide polymorphisms identifier as recorded in the Single Nucleotide Polymorphism Database (dbSNP), *SCV*# novel variants have been registered in the database of genotype–phenotype associations ClinVar, *MAF* minor allele frequency, *New* new variant detected in this study, *NA* not available

^a^Age of proband at last clinical evaluation

^b^In italics, classified in this study according to ACMG Standards and Guidelines; normal, ClinVar last evaluation; VUS, variant of uncertain significance

^c^Best’s disease stages of left (LE) and right eye (RE) as diagnosed in probands at the age of last clinical evaluation (stages 1 to 5)

^d^BCVA (best-corrected visual acuity) in logMAR scale (logarithm of Minimum Angle of Resolution), logMAR 0/0 (LE/RE) indicates standard vision, logMAR > 0.5/0.5 indicates low vision, logMAR > 1.3/1.3 m indicates blindness

* Family segregation analysis

## Results

### Genotype

The subjects of the study were 30 males and 27 females (mean age ± SD 40.7 ± 18.6 years; range 9-85) with clinical features suggesting Best’s disease. Genetic testing revealed 26 different variants in 36 patients who tested positive (63% positivity); 20 variants are already known to be associated with Best disease, and six were novel and associated with BVMD for the first time (Table 1). One of the six new variants regards a nucleotide substitution that causes an amino acid change already established as pathogenic, and we therefore do not discuss it here. We describe the clinical features of probands carrying the other five new variants in Additional files 1, 2.

**Fig. 3:**
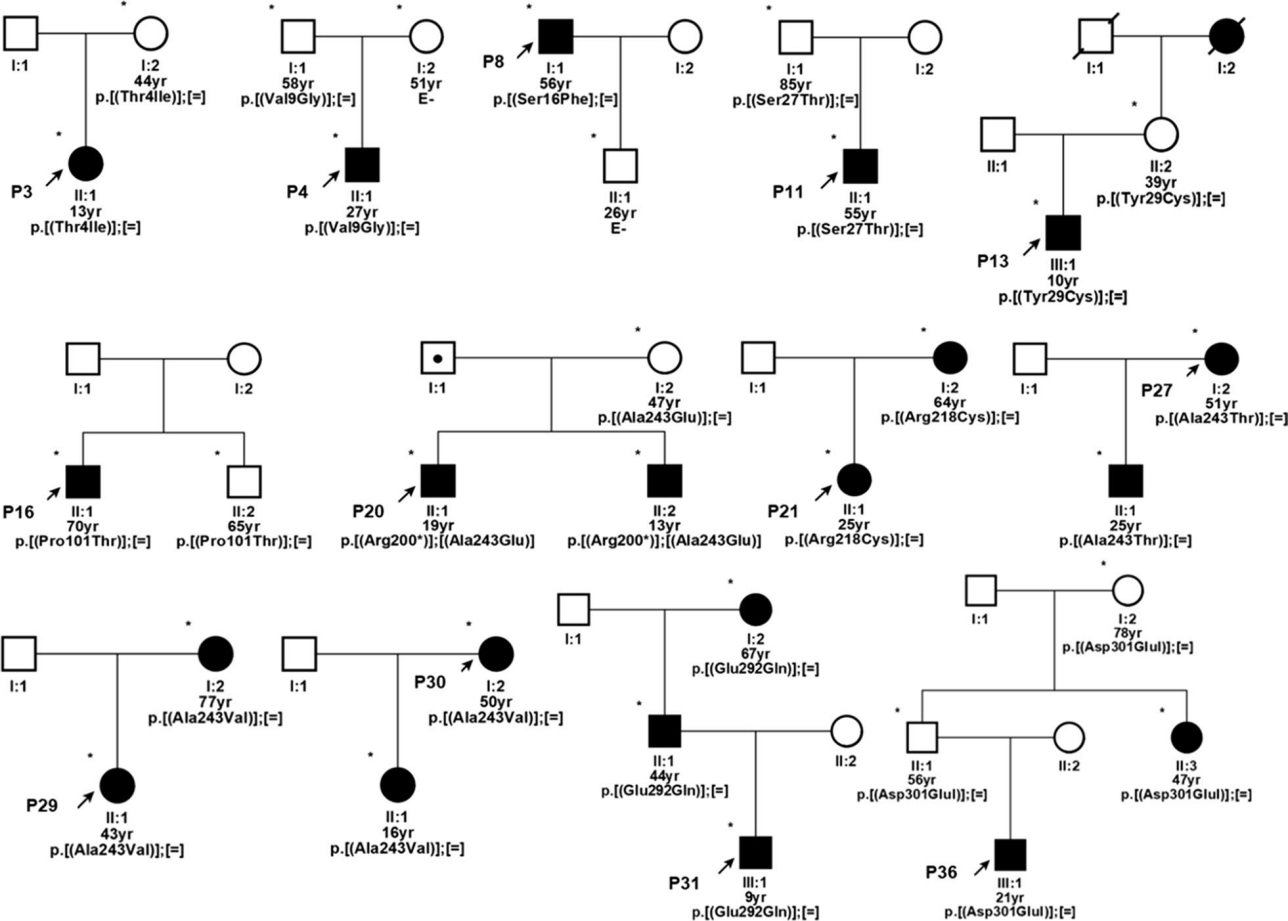
Pedigrees of 13 probands with Best disease. P, proband; E−, negative to genetic test; square, male subject; circle, female subject; black symbol, affected subject; white symbol, healthy subject; * documented clinical evaluation; ● obligate carrier; yr, age in years; maternal and paternal alleles are identified by square brackets; =, indicates no change in the second protein allele

Some family members of 13 patients testing positive for a variant in *BEST1* were also studied by target sequencing: 16 out of 18 carried a variant in *BEST1*, eight of whom were clinically healthy and eight affected (Fig. 3). Penetrance in our families was estimated at 72.4% with no sex differences (71.4% and 73.3% for females and males, respectively).

Extension of study to family members was only possible for 2 out of 6 new variants: p.(Val9Gly) found in the asymptomatic father of proband 3, and p.(Asn296Lys) found in the affected father and grandmother of proband 31 (Fig. 3).

**Fig. 4:**
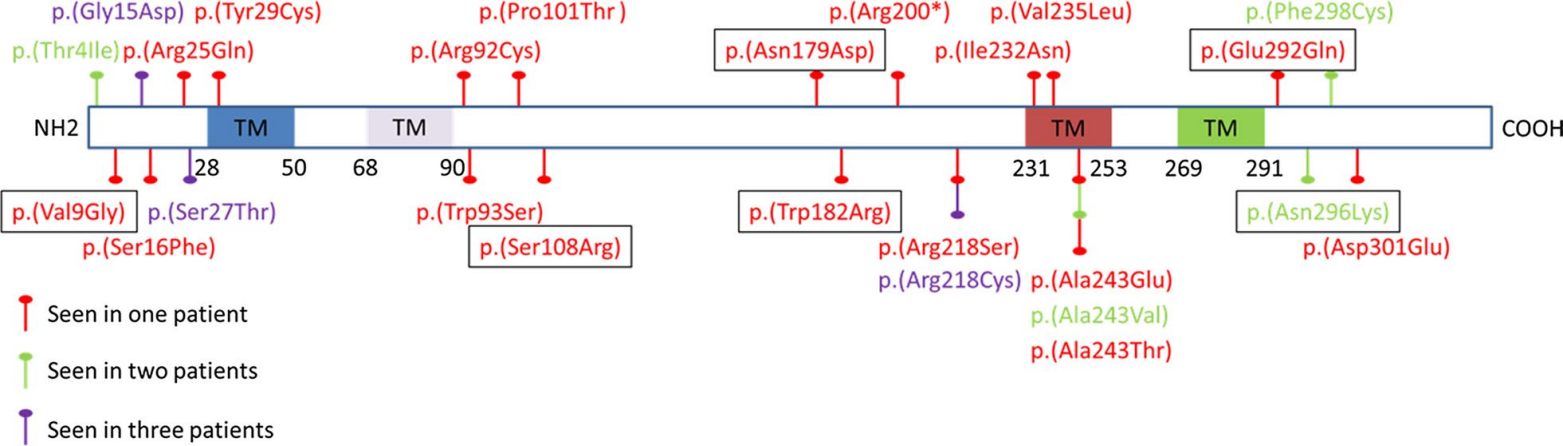
Distribution of *BEST1* variants found in our patients. Graphical representation of the distribution of *BEST1* variants throughout the gene. New variants are shown in rectangular boxes

Pathogenic *BEST1* variants were scattered throughout the gene (Fig. 4).

### Structural biology of *BEST1* variants

**Fig. 5:**
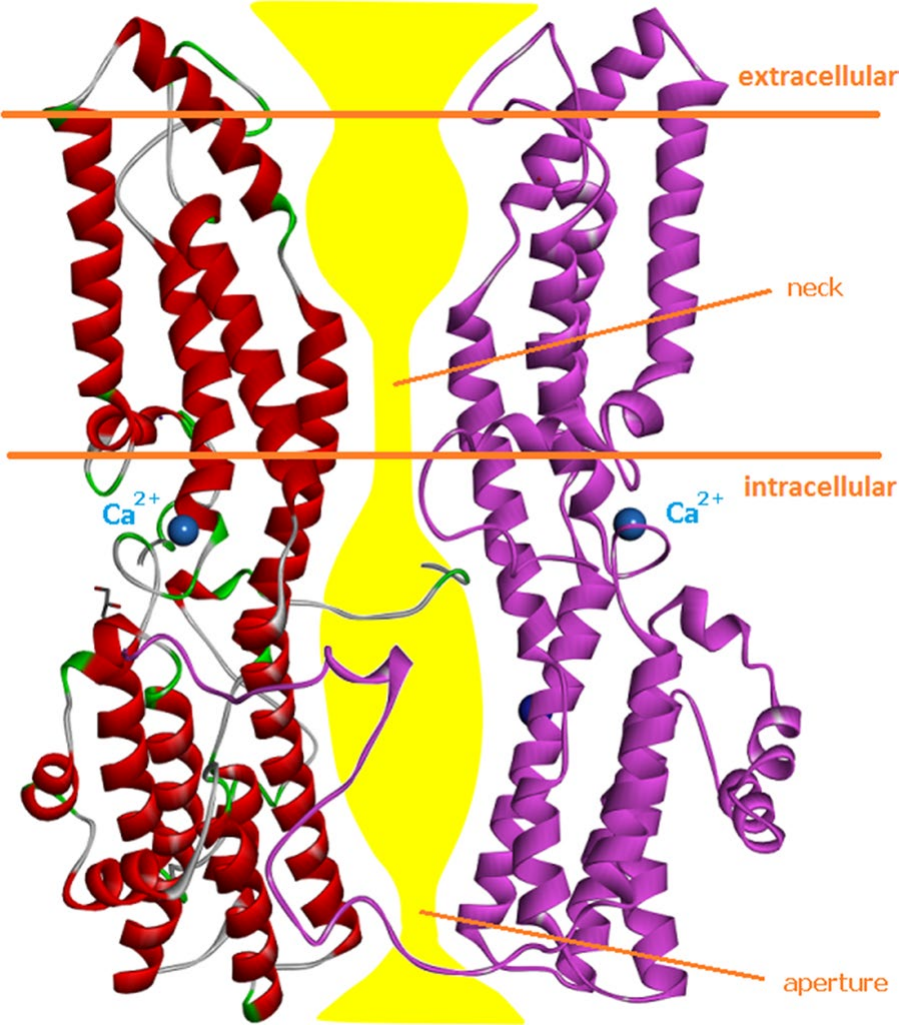
Subunits C and E of the X-ray structure of chicken bestrophin-1 (in ribbon representation) forming part of the calcium-activated chloride channel [9, 10]. The axial cross-section of the ion pore (yellow tube) shows the approximate size and shape of the pore and indicates the positions of the pore neck and aperture as well as calcium binding sites involved in channel gating. Boundaries of the cell membrane are indicated by the horizontal red lines (figure adapted from: Ref. [10])

**Fig. 6:**
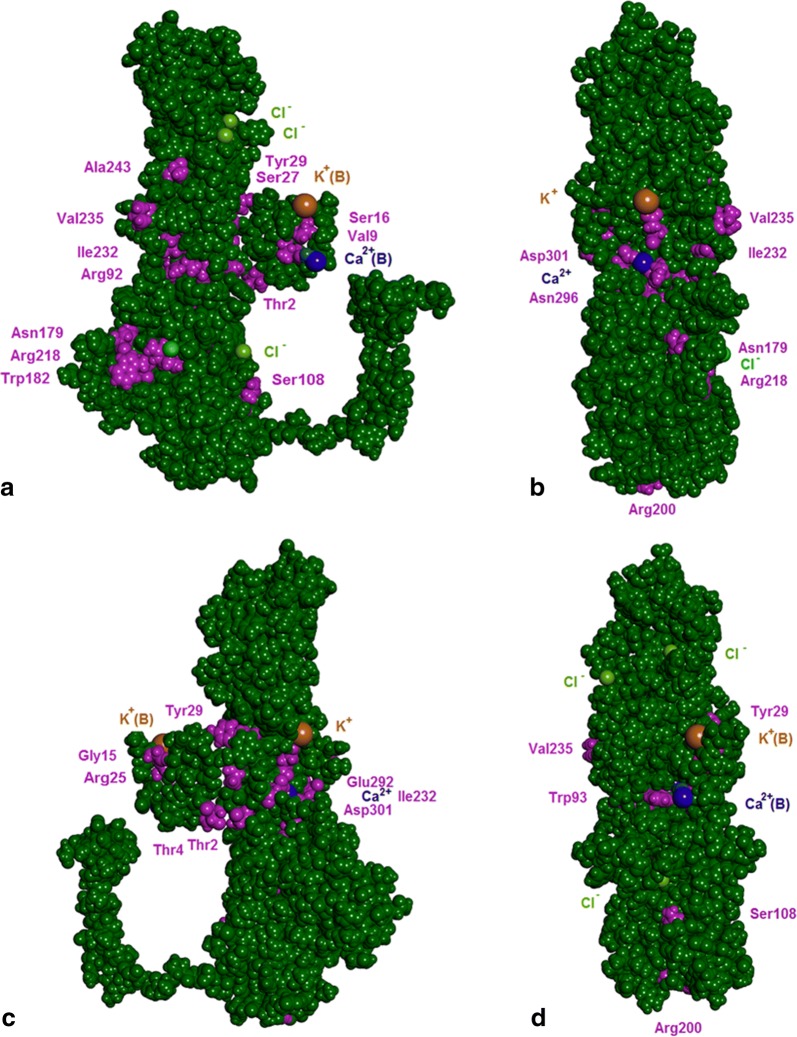
3D model of subunit A of human bestrophin-1 (residues 1-366; CPK representation) prepared by homology modelling and relaxed by geometrical optimization in water by molecular mechanics from crystal structure of chicken BEST1 [9, 10]. Ca^2+^ and K^+^ ions of neighbouring subunit B in the chloride channel coordinated by the C-terminal domain residues of subunit A are marked with (B). The locations of individual variant amino acids identified in the probands (Table 1) are shown in purple. **a** Front view of subunit A with “tail” wrapped around subunit B (channel pore inner side). **b** Side view (binding site to neighbouring subunit E). **c** Rear view (membrane side). **d** Side view (binding site to neighbouring subunit B)

A variant can affect the structure and function of BEST1 CaCC in a number of ways. Depending on its site in bestrophin-1 molecular structure, an amino acid replacement can lead to: (i) altered Ca^2+^ binding and defective channel activation; (ii) conformational changes affecting channel gating; (iii) anomalous binding between bestrophin-1 subunits and modified channel stability; (iv) variations in channel pore size and shape and altered ion throughput rate or specificity; and (v) other changes (Fig. 5). We studied structural changes linked to the 36 variants (Table 2) in a 3D model of human bestrophin-1 prepared by comparative modelling from the crystal structure of chicken BEST1 [9, 10]. The locations of the amino acid variants in the 3D structure of bestrophin-1 of the probands are shown in Fig. 6.

**Table 2:** Computational biology characterization of detected variants

Proband ID^a^	Mean BDSI^b^ [%]	Variant	Location of variant residue in human BEST1 3D structure	Possible structural consequences of residue replacement	Relative energy of dimerization ΔΔE_dim_^c^ [kcal mol^−1^]	Relative energy of Ca^2+^ binding ΔΔE_Cabin_^d^ [kcal mol^−1^]	PMVA predicted pathogenicity of *BEST1* variant in logMAR scale^e^
P1	11.90	p.(Thr2Ile)	At interface to subunit B of cacc Close to K^+^ binding site of subunit B Close to Ca^2+^ binding site of subunit B	Channel formation and stability Channel pore shape Channel activation	− 23	− 91	0.24
P2, P3	3.55	p.(Thr4Ile)	At interface to subunits B and E of cacc Close to K^+^ binding site of subunit B Close to Ca^2+^ binding site of subunit B	Channel formation and stability Channel pore shape Channel activation	− 13	9	0.19
P4	17.60	p.(Val9Gly)	At interface to subunit B of cacc Close to K^+^ binding site of subunit B Close to Ca^2+^ binding site of subunit B	Channel formation and stability Channel pore shape Channel activation	− 34	62	0.29
P5–P7	7.10	p.(Gly15Asp)	At interface to subunit B of cacc Close to K^+^ binding site of subunit B Close to Ca^2+^ binding site of subunit B	Channel formation and stability Channel pore shape Channel activation	− 1	4	0.13
P8	17.60	p.(Ser16Phe)	At interface to subunit B of cacc Close to K^+^ binding site of subunit B Close to Ca^2+^ binding site of subunit B	Channel formation and/or pore shape Channel stability Channel activation	50	− 19	0.37
P9	15.30	p.(Arg25Gln)	At interface of cacc to cell membrane Close to K^+^ binding site of subunit B Close to Ca^2+^ binding site of subunit B	Channel embedding in cell membrane Channel pore shape and stability Channel activation	11	− 49	0.18
P10–P12	23.10	p.(Ser27Thr)	At interface to subunit B of cacc Close to K^+^ binding site of subunit B Close to Ca^2+^ binding site of subunit B	Channel formation and stability Channel pore shape Channel activation	− 71	16	0.47
P13	0.00	p.(Tyr29Cys)	At interface to subunit B of cacc Close to K^+^ binding site of subunit B Close to Ca^2+^ binding site of subunit B	Channel formation and stability Channel pore shape Channel activation	− 13	− 3	0.19
P14	NA	p.(Arg92Cys)	Lining pore wall of cacc Close to Ca^2+^ binding site of subunit A At interface to subunit E of cacc	Conformational changes, channel gating Chloride ion throughput or ion selectivity Channel activation Channel stability	12	− 23	0.19
P15	NA	p.(Trp93Ser)	Close to pore wall of cacc Close to Ca^2+^ binding site of subunit A At interface to subunit B of cacc	Conformational changes, channel gating Chloride ion throughput or ion selectivity Channel activation Channel stability	12	− 15	0.19
P16	9.50	p.(Pro101Thr)	Lining pore wall of cacc Near interface to subunit B of cacc	Conformational changes, channel gating Chloride ion throughput or ion selectivity Channel stability	− 17	− 24	0.21
P17	32,60	p.(Ser108Arg)	At interface to subunit B of cacc	Channel formation and/or pore shape Channel stability	96	− 7	0.59
P18	15.30	p.(Asn179Asp)	At interface to subunits D and E of cacc	Channel formation and/or pore shape Channel stability	23	− 50	0.24
P19	NA	p.(Trp182Arg)	At interface to subunit E of cacc	Channel formation and/or pore shape Channel stability	26	25	0.25
P20	NA	p.(Arg200*)	Cytoplasm side of cacc	Channel gating	–	–	–
P21–P23	7.60	p.(Arg218Cys)	At interface to subunit E of cacc Near Cl^−^ binding site	Channel stability Channel formation and/or pore shape	− 1	25	0.13
P24	23.40	p.(Arg218Ser)	At interface to subunit E of cacc Near Cl^−^ binding site	Channel stability Channel formation and/or pore shape	32	55	0.28
P25	31.40	p.(Ile232Asn)	At interface to subunit E of cacc Near pore wall of cacc Close to Ca^2+^ binding site of subunit A	Channel stability Conformational changes, channel gating Chloride ion throughput or ion selectivity Channel activation	21	− 46	0.23
P26	6.50	p.(Val235Leu)	At interface to subunit E of cacc	Channel formation and stability	6	7	0.16
P27	46.20	p.(Ala243Thr)	At interface to subunit E of cacc	Channel formation and stability	5	− 47	0.15
P28–P30	6.50	p.(Ala243Val)	At interface to subunit E of cacc	Channel formation and stability	− 7	− 48	0.16
P31	70.30	p.(Glu292Gln)	At Ca^2+^ binding site of subunit A Close to K^+^ binding site of subunit A At interface to subunit E of cacc	Channel activation Channel stability Channel formation and stability	− 14	42	0.20
P32, P33	12,85	p.(Asn296Lys)	At Ca^2+^ binding site of subunit A At interface to subunit E of cacc	Channel activation Channel formation and stability	− 17	80	0.21
P34, P35	NA	p.(Phe298Cys)	Near Ca^2+^ binding site of subunit A Near interface to subunit E of cacc	Channel activation Channel formation and stability	26	− 35	0.25
P36	18.70	p.(Asp301Glu)	At Ca^2+^ binding site of subunit A At interface to subunit E of cacc At interface of cacc to cell membrane	Channel activation Channel formation and stability Channel embedding in cell membrane	− 60	− 7	0.42

^a^Probands bearing the same amino acid replacement are grouped together

^b^Mean age-adjusted Best’s Disease Severity Index (%BDSI) of probands bearing the same bestrophin-1 variant as defined in Eq. (5). In our cohort the number of probands sharing the same variant ranges from 1 to 3. On the logMAR scale, logMAR_LE,i_ + logMAR_RE,I_ = 0 for a person with standard vision and 2.6 for a completely blind proband. Examples of BDSI index: BDSI(logMAR_LE_, logMAR_LE_, Age) = BDSI (0, 0, 100) = 0% means no Best’s disease symptoms during whole lifetime (100 years); BDSI(1.3, 1.3, 1) = 100% means the most severe Best’s disease symptoms—complete (100%) loss of vision from early childhood; BDSI (0.4, 0.6, 40) = 26% means relatively mild Best’s disease symptoms (26% loss of vision) at age 40 years

^c^Relative energy of human BEST1 dimerization ΔΔE_dim_ estimates the extent of damage to CaCC as the change in binding energy between two neighbouring bestrophin-1 subunits A and B in CaCC caused by a variant, as compared to the reference native protein BEST1, see Eqs. (1) and (2), Methods

^d^Relative Ca^2+^ binding energy ΔΔE_Cabin_ estimates quantitative damage to channel regulation expressed as altered ability of BEST1 dimer AB to bind calcium ions caused by a variant, compared to the reference native protein BEST1, see Eqs. (3) and (4) Methods

^e^Pathogenicity of a given *BEST1* variant: PMVA—Predicted Mean Visual Acuity (logMAR_LE_ + logMAR_RE_)/2 of an individual with a given bestrophin-1 variant at age 40 years in the logMAR scale. The PMVA calculated from Eq. (7) is based on the modulus of computed relative energy of variant BEST1 subunit dimerization |ΔΔE_dim_| with respect to native human BEST1 and regression equation of the QSAR model of BEST1 dimerization: BDSI = 0.24935·|ΔΔE_dim_| + 6.56527 (Fig. 7)

Besides locating variant residues, we used molecular mechanics to calculate the relative energies of dimerization (ΔΔE_dim_, Eqs. 1 and 2) of two bestrophin-1 subunits bearing a variant. The dimerization energies approximate the probability that assembled bestrophin-1 variants display an alteration of the ideal quaternary structure of CaCC affecting its function. We also computed relative energies of calcium binding (ΔΔE_Cabin_, Eqs. 3 and 4) by the variant bestrophin-1 subunits as an approximation of the possibility of altered calcium-dependent regulation of CaCC for all the variants detected in the probands (Table 2). The relative energies therefore characterize deviation from native bestrophin-1 structure and/or function, presumably reflecting potential pathogenicity.

To explore the possibility of a link between the computed energies (ΔΔE_dim_, ΔΔE_Cabin_ and |ΔΔE_dim_|) and clinical symptoms of probands with the *BEST1* variants in question, we established a QSPR [29]. In the QSPR model we used the age-adjusted mean Best’s Disease Severity Index (BDSI), averaged over all N probands with the same variant, to represent the severity of observed clinical symptoms on a quantitative scale (0% to 100%):

5 $${\text{BDSI}} = \frac{1}{\text{N}}\mathop \sum \limits_{\text{i}}^{\text{N}} \left[ {\frac{{{\text{logMAR}}_{{{\text{LE}},{\text{i}}}} + {\text{logMAR}}_{{{\text{RE}},{\text{i}}}} }}{2.6} \cdot 100{\text{\% }} \cdot {\text{e}}^{{ - \frac{{{\text{A}}_{\text{i}} }}{100}}} } \right]$$

The summation in Eq. (5) includes probands with the same variant, where logMAR_LE,i_ and logMAR_RE,i_ are left (LE) and right eye (RE) best-corrected visual acuities (BCVA) of probands in the log Minimum Angle of Resolution scale (logMAR—Table 1) determined by clinical evaluation. In Eq. (5), the sum of BCVA was normalized by a factor of 2.6 to express loss of vision as a percentage (logMAR = 0/0 means perfect vision of LE/RE, while logMAR ≥ 1.3/1.3 means complete blindness of both eyes). In Eq. (5), A_i_ is the age of proband i at the time of clinical evaluation and the corresponding time factor of the BDSI: ${\text{f}} = {\text{e}}^{{ - \frac{{{\text{A}}_{\text{i}} }}{100}}}$ adjusts the severity index for age of disease onset (${\text{f}} \cong 1$ for a patient diagnosed at age ${\text{A}}_{\text{i}} = 1$ year while ${\text{f}} \cong 0.368$ for a patient diagnosed with BVMD at age ${\text{A}}_{\text{i}} = 100$ years). BDSI = 0% means perfect vision (no symptoms), BDSI = 100% means complete loss of vision. See legend of Table 2 for examples of BDSI index values in relation to BCVA and age of proband. The BDSI was analyzed for correlations with computed energies (ΔΔE_dim_, ΔΔE_Cabin_) and with |ΔΔE_dim_|. The modulus of dimerization energy was used in the QSPR models because we assumed that elevated and low relative energies of bestrophin-1 subunit dimerization could both be harmful to the normal structure and function of the CaCC.

**Fig. 7:**
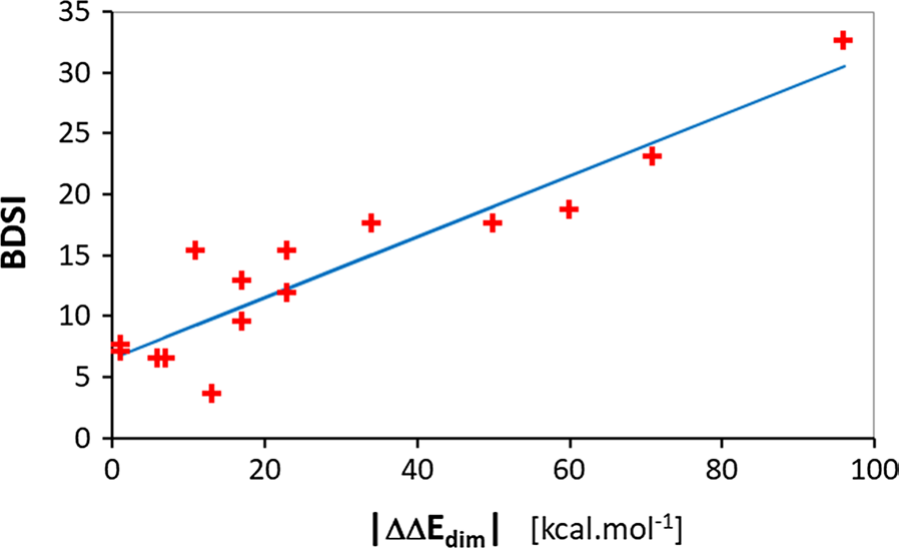
Plot of regression equation of Best Disease Severity Index (BDSI) versus modulus of relative energy of dimerization |ΔΔE_dim_| of variant BEST1 subunits: BDSI = 0.24935 · |ΔΔE_dim_| + 6.56527, Eq. (6). BDSI was derived from clinically evaluated BCVA (best-corrected visual acuity in logMAR scale) and age of 27 probands, Eq. (5), Table 2; ΔΔE_dim_ was computed by molecular mechanics from the 3D model of human CaCC. Statistical parameters of the correlation: number of available data points: n = 20, squared correlation coefficient: R^2^ = 0.854, leave-one-out cross-validated correlation coefficient: ${\text{R}}^{ 2}_{\text{xv}}$ = 0.827, Fischer F-test: F = 76.164, significance of correlation: α > 95%, number of outliers removed: n_o_ = 5

We managed to establish a statistically significant QSPR model correlating BDSI with the modulus of computed relative dimerization energy:

6 $${\text{BDSI}} = 0.24935 \cdot \left| {\Delta \Delta {\text{E}}_{ \dim } } \right| + 6.56527$$

(for details see Fig. 7) and linking structural biology considerations to the clinical findings. This validated QSPR model also enables prediction of clinical symptoms (predicted mean visual acuity averaged over both eyes at a given patient age, PMVA or logMAR_ave_) of other similar *BEST1* variants and estimation of the pathogenicity of *BEST1* variants based on |ΔΔE_dim_| computed for a given variant by molecular mechanics:

7 $${\text{PMVA}} = {\text{logMAR}}_{\text{ave}} = \frac{2.6}{200} \cdot \left[ {0.24935 \cdot \left| {\Delta \Delta {\text{E}}_{ \dim } } \right| + 6.56527} \right] \cdot {\text{e}}^{{ \frac{\text{A}}{100}}}$$

where A is the age of the patient (set at 40 years in our predictions, Table 2). In the logMAR scale (0 to 1.3 or more) higher values mean worse visual function, so that *BEST1* variants leading to predicted logMAR_ave_ values higher than 0.5 (corresponding to |ΔΔE_dim_| ≥ 77 kcal mol^−1^) can be classified as potentially pathogenic. Calculated logMAR_ave_ values lower than 0.5 are regarded as less likely to be pathogenic (Table 2). The threshold 0.5 is based on the WHO criteria for low vision using the logMAR scale [31].

Finally, out of the six new *BEST1* variants reported here, four residue replacements [p.(Val9Gly), p.(Asn179Asp), p.(Glu292Gln) and p.(Asn296Lys)] seem to affect Ca^2+^ ion binding and CaCC activation (Table 2).

## Discussion

Computational modelling of the tertiary structure of bestrophin-1 and quaternary structure of CaCC made it possible to link the detected variants to possible molecular mechanisms that may be involved in channel function impairment. In relation to the location of individual amino acid replacements in the 3D structure of bestrophin-1, the variants may affect channel shape, stability, activation, gating, ion selectivity, ion throughput, and probably other features as well. Our computational structural biology approach was therefore based on characteristics involving protein structure and function which are more complex than similarity/diversity of variant amino acid residues.

It is complicated to quantify the clinical features of probands with Best vitelliform macular dystrophy. Best disease stages of the left and right eye as diagnosed in our probands were not correlated with BCVA in the logMAR scale (Table 1). We, therefore, selected BCVA and designed our own age-adjusted quantitative Best’s disease severity index (BDSI), see Eq. 5. The BDSI index showed a good correlation with the computed modulus of bestrophin-1 subunit dimerization energy |ΔΔE_dim_| (Fig. 7) which is assumed to reflect the potential harmful effect of individual missense variants on *BEST1* CaCC structure and function. During regression analysis, it was necessary to remove five outlier points (probands P13, P24, P25, P27 and P31) identified by the leave-one-out cross-validation algorithm. The corresponding point variants (p.(Tyr29Cys), p.(Arg218Ser), p.(Ile232Asn), p.(Ala243Thr) and p.(Glu292Gln) are mainly residue replacements leading to low-to-medium positive and negative ΔΔE_dim_ values, which are thus expected to be linked to small changes in CaCC structure and function. We can therefore assume that the QSPR model and the proposed computational tool for *BEST1* variant pathogenicity prediction apply to conservative as well as radical variants. Although many intrinsic factors are known to co-determine the biological consequences of gene diversity and the resulting impairment of health or well-being [32], we were able to establish a robust but simple QSPR model linking computed relative energies of human bestrophin-1 protein with the symptoms of BVMD diagnosed in a cohort of patients. To illustrate application of the model we compare the PMVA of two probands, P4 and P17, of similar age bearing different *BEST1* variants and displaying different clinical symptoms (Table 3). As we can see, patient P17 has greater visual impairment (higher logMAR_ave_ and mean BDSI values). Structural biology assessment of his *BEST1* variant predicts larger alterations of stability and structure of assembled bestrophin-1 subunits (larger |ΔΔE_dim_|), possibly detrimental to CaCC function. Consequently, predicted mean visual acuity (PMVA) estimated for P17 at age 40 years is larger than the threshold of 0.5 (PMVA = 0.59) and the corresponding *BEST1* variant p.(Ser108Arg) can thus be considered likely pathogenic.

**Table 3:** Comparison of two *BEST1* variants

Proband ID	*BEST1* variant	Age^a^ [years]	BCVA_LE_^b^	BCVA_RE_^b^	logMAR_ave_^c^	Mean BDSI^d^ [%]	|ΔΔE_dim_|^e^ [kcal mol^−1^]	PMVA^f^	Estimated *BEST1* variant pathogenicity
P4	p.(Vla9Gly)	27	0.0	0.6	0.30	17.6	34	0.29	Likely non-pathogenic
P17	p.(Ser108Arg)	26	0.1	1.0	0.55	32.6	96	0.59	Likely pathogenic

^a^Age of proband at last clinical evaluation

^b^BCVA (best-corrected visual acuity) in logMAR scale of left (LE) and right eye (RE)

^c^Average BCVA value (logMAR_LE_ + logMAR_RE_)/2

^d^Mean age-adjusted Best’s Disease Severity Index

^e^Modulus of computed relative energy of variant BEST1 subunit dimerization

^f^PMVA—predicted mean visual acuity (logMAR_LE_ + logMAR_RE_)/2 of an individual with a given bestrophin-1 variant at age 40 years

Further calibration of this computational pathogenicity estimate on a much larger cohort of patients and wider range of amino acid variants is still needed to set the borderline between low and high pathogenicity and confirm the reliability of the resulting clinical outcome predictions.

## Conclusions

Similar computational biology approaches based on molecular mechanics energies or other structural properties of native and variant proteins and their interactions suggest the possibility of exploring the molecular basis of genetic diseases and perhaps predicting likely clinical outcomes of genetic variants. Worth to note, the predicted pathogenicity descriptor PMVA can also be used for estimating the development of patient’s BCVA over the years by substituting selected age (A) into Eq. (7).

We also reported the results of genetic testing in 57 Italian BVMD patients, including 36 subjects with a *BEST1* variant. Compared with other reports in the literature for different populations, our results indicate that there are no specific ethnic variants [2], while segregation study confirmed the variable penetrance and expressivity of the disease in patients with the same *BEST1* variant, even in the same family [33, 34]. Six new variants are also reported, thus broadening the *BEST1* variant spectrum.

## Supplementary information

**Additional file 1.** Clinical findings for patient P4, P17, P18, P19 and P31

**Additional file 2.** **Figure S1-P4**: Right eye five-year follow-up in a 22-year-old patient carrying a p.Val9Gly variant in BEST1. SD-OCT scans and corresponding FAF through the fovea and vitelliform deposit at baseline (A, B and C) and 5 years later (D, E and F). After 5 years the patient showed a “vitelliruptive” stage with irregular FAF signal (D), elongation of the outer segments under the fovea (E) and a thin neurosensory retinal detachment surrounding the lipofuscin deposit (E, F). Fundus photograph of the posterior pole at the last follow-up (G). **Figure S2-P4**: Left eye five-year follow-up of the same patient. At baseline a “pseudohypopyon” stage is clearly visible in FAF (A) and OCT scans (B, C). Serous detachment of the neurosensory retina is visible in the upper macula with decreased FAF levels (Fig. 2B). In the bottom row of images, the yellow vitelliform material is evident by FAF. Five years later, there was reduction of the serous detachment and contraction of the deposit (“vitelliruptive” stage). Irregular FAF signal (D), preserved elongation of photoreceptors (E, F) and fundus photograph of posterior pole in “vitelliruptive” stage (G). **Figure S3-P17**: FAF images of Best’s maculopathy patient (P17) (A), showing a lesion consisting of a circular hyperautofluorescent parafoveal circle with an annular hypofluorescent ring enclosing a second inner hyperautofluorescent ring and a hypoautofluorescent central spot (tip of the dome). The infrared images show hyperreflective foveal changes more visible in the LE (B bottom). The corresponding SD-OCT scans in both eyes (C) show subfoveal hyperreflective lipofuscin deposits at RPE level, surrounded by thin neurosensory retinal detachment, more prominent in the LE. The photoreceptor layer, partially intact in the RE and with slight disruption in the LE, is displaced on top of the lesion. In the LE tiny microcystic spaces subtending inner-layer retinal splitting can also be observed. **Figure S4-P18**: FAF (A, C) and OCT scans (B, D) of a 46-year-old patient carrying a p.Asn179Asp variant in BEST1. Idiopathic choroidal folds and central accumulation of hyperautofluorescent lipofuscin is evident in both eyes. The right eye is in “pseudohypopyon” stage, as suggested by the neurosensory retinal detachment, while the left eye is still in “vitelliform” stage. **Figure S5-P19**: FAF and OCT scans of a 13-year-old girl with a Trp182Arg variant in BEST1, showing bilateral vitelliform lesions at pseudohypopyon stage in both eyes. OCT scans show subfoveal hyperreflective lipofuscin deposits at RPE level, surrounded by thin neurosensory retinal detachment. **Figure S6-P31**: Patient 31 (P31), a 9-year-old boy, showing fundus ophthalmoscope evidence of bilateral circular yellowish yolk-like lesions in the macular area (D). The lesions are symmetrically hyperautofluorescent with a nasal hypoautofluorescent sickle visible by FAF (A). At the same location in IR images, there is a circular area of hyporeflectivity matching the lipofuscin deposit at sub-RPE level with consequent reorganization of the IS-OS junction layer on top of the dome, as seen also in OCT scans (C). Preservation of the photoreceptor layer is also confirmed by adaptive optical images, showing cone mosaic structure in the RE (E), where the lesion appears elevated but with a normal arrangement of cones on top of it. In fact, intact bright cones can be seen across the central 6 degree eccentricity around the fovea, as well as a circular dark ring of shadow delineating the contour line of the lesion, where faint, still resolvable cones seem mechanically distorted.

## Data Availability

The datasets used and/or analyzed during the current study are available from the corresponding author on reasonable request.

## Abbreviations

ADVIRC: autosomal dominant vitreoretinochoroidopathy
AO: adaptive optics
ARB: autosomal recessive bestrophinopathy
BCVA: best-corrected visual acuity
BDSI: Best’s Disease Severity Index
B-FAF: blue fundus autofluorescence
BVMD: best vitelliform macular dystrophy
CaCC: calcium-activated chloride channel
ΔΔE_dim_: subunit dimerization energy
ΔΔE_Cabin_: calcium binding energy
EOG: electro-oculogram
IR: infra-red
LE: left eye
mfERG: multifocal electroretinogram
PMVA: predicted mean visual acuity
QSPR: quantitative structure-pathogenicity relationship
RE: right eye
RP: retinitis pigmentosa
RPE: retinal pigment epithelium
SD-OCT: spectral domain optical coherence tomography
