# Supplementary material for: Pathogenicity of new BEST1 variants identified in Italian patients with best vitelliform macular dystrophy assessed by computational structural biology
Source: J Transl Med. 2019 Oct 1;17:330. doi: 10.1186/s12967-019-2080-3 (PMC6771118; doi:10.1186/s12967-019-2080-3)
Supplement: Supplementary file 1 — Additional file 1. Clinical findings for patient P4, P17, P18, P19 and P31 [file 12967_2019_2080_MOESM1_ESM.docx]

**Clinical findings**

***Patient 4, p.(Val9Gly)***

The new p.(Val9Gly) variant was identified in a 27-year-old male (P4) with a BVMD phenotype. At the first evaluation at age 22 years, visual acuity with the ETDRS chart was 20/80 and 20/20 in right and left eyes (RE and LE), respectively. The disease was at different stages in the two eyes: RE posterior pole findings were consistent with Best disease in “vitelliform” stage (Fig. S1-A), while LE was already at “pseudohypopyon” stage (Fig. S2-A). The RE showed a central subfoveal deposit, presumably lipofuscin, as suggested by the corresponding increased FAF signal (Fig. S1-A). OCT scan showed an apparently intact external limiting membrane, while the ellipsoid and interdigitation zone bands had an irregular pattern (Fig. S1-B). The LE showed a central area with two levels: in the upper part there was serous detachment of the neurosensory retina with corresponding lower FAF levels (Fig. S2-B), whereas in the lower part, there was still yellow, vitelliform material with increased FAF signal (Fig. S2-C).

At five-year follow-up, both eyes progressed to “vitelliruptive” stage: the yellow lipofuscin material broke up, determining a mottled FAF signal. Indeed FAF images showed focal spots with increased and decreased signal (Fig. S1-D, Fig. S2-D) due to atrophic degeneration of the outer retinal layers. Right eye OCT scans showed progressive contraction of the material into a thick, highly reflective, yellowish deposit surrounded by thin neurosensory retinal detachment between the highly reflective bands of the RPE and the ellipsoid zone (Fig. S1-E, S1-F; Fig. S2-E, S2-F). Left eye OCT still showed retinal detachment with shedding of photoreceptors and a focal deposit of material nasally with respect to the fovea (Fig. S2-F). At this stage, visual acuity was 20/20 in both eyes.

***Patient 17, p.(Ser108Arg)***

Patient 17 (P17), a 26-year-old male with a history of juvenile maculopathy, showed almost normal visual acuity (BCVA 20/20 RE, 20/25 LE) and high hyperopic defect (+8 sphere in both eyes). Fundus examination detected a yellowish bilateral dome-shaped lesion in the inferior perifoveal area surrounded by diffuse RPE abnormalities. FAF (Fig. S3-A) showed the lesion as a circular parafoveal hyperautofluorescent circle, with an annular hypofluorescent ring enclosing a second inner hyperautofluorescent ring and an hypoautofluorescent central spot (tip of dome). The infrared hyperreflective foveal change was more prominent in LE (Fig. S3-B). The corresponding SD-OCT scans in both eyes (Fig. S3-C) showed remarkable modification of foveal profile due to subfoveal hyperreflective lipofuscin deposits at RPE level, surrounded by mild adjacent neurosensory retinal detachment. The photoreceptor layer seemed displaced on top of the lesion, partially intact in RE and slightly disrupted in LE, where tiny microcystic spaces, subtending inner layer retinal splitting, were also observed. Multifocal electroretinograms only showed reduced response amplitude densities from the central area (0-2.5° of foveal eccentricity) of both eyes, RAD being within normal limits in other external areas. At 12-month follow-up, morphological and functional data was substantially unmodified.

***Patient 18, p.Asn179Asp***

Variant p.(Asn179Asp) was identified in this 46-year-old male (P18) with different stages of BVMD in the two eyes. OCT scans of both eyes showed idiopathic choroidal folds and central lipofuscin deposits (Fig. S4-A, S4-C), although RE showed serous neurosensory retinal detachment with shedding of photoreceptors, suggesting a “pseudohypopyon” stage of the disease (Fig. S4-B), while LE was still in “vitelliform” stage. BCVA was 20/40 in both eyes. The patient was unfortunately lost to follow-up.

***Patient 19, p.(Trp182Arg)***

Patient 19 (P19), a 13-year-old girl, referred with a diagnosis of suspected hereditary macular dystrophy, presented with a visual acuity of 0.6 in both eyes. She showed bilateral vitelliform lesions at pseudohypopyon stage (Fig. S5). Follow-up examination one year later did not show any change in the clinical picture.

***Patient 31, p.(Glu292Gln)***

Patient 31 (P31), a 9-year-old boy with a family history of BVMD (affected father and grandmother, who also underwent segregation analysis), was referred for bilateral maculopathy and normal visual acuity in both eyes (BCVA was 20/20 with no refractive error). Fundus examination showed bilateral circular yellowish yolk-like lesions (Fig. S6-D) that were hyperautofluorescent with a nasal hypoautofluorescent sickle by FAF (Fig. S6-A). IR images showed a circular hyporeflective foveal lesion coinciding with symmetrical lipofuscin deposits at sub-RPE level with consequent reorganization of the IS-OS junction layer on top of the dome (Fig. S6-C). This can also be appreciated in AO images at photoreceptor level showing cone mosaic structure in RE (Fig. S6-E). Intact bright cones can be seen across the central 6-degree eccentricity around the fovea, as well as a circular dark ring delineating the contour line of the lesion, where faint, still resolvable cones seem mechanically distorted. Although elevated, the lesion showed a normal arrangement of cones on top of it. This may be why photoreceptor function was preserved, as indicated by normal BCVA and mfERG RAD values. At 12-month follow-up, this young patient did not show any remarkable change in retinal structure or function.
