# Supplementary material for: Pathogenicity of new BEST1 variants identified in Italian patients with best vitelliform macular dystrophy assessed by computational structural biology
Source: J Transl Med. 2019 Oct 1;17:330. doi: 10.1186/s12967-019-2080-3 (PMC6771118; doi:10.1186/s12967-019-2080-3)
Supplement: Supplementary file 2 — Additional file 2. Figure S1-P4: Right eye five-year follow-up in a 22-year-old patient carrying a p.Val9Gly variant in BEST1. SD-OCT scans and corresponding FAF through the fovea and vitelliform deposit at baseline (A, B and C) and 5 years later (D, E and F). After 5 years the patient showed a “vitelliruptive” stage with irregular FAF signal (D), elongation of the outer segments under the fovea (E) and a thin neurosensory retinal detachment surrounding the lipofuscin deposit (E, F). Fundus photograph of the posterior pole at the last follow-up (G). Figure S2-P4: Left eye five-year follow-up of the same patient. At baseline a “pseudohypopyon” stage is clearly visible in FAF (A) and OCT scans (B, C). Serous detachment of the neurosensory retina is visible in the upper macula with decreased FAF levels (Fig. 2B). In the bottom row of images, the yellow vitelliform material is evident by FAF. Five years later, there was reduction of the serous detachment and contraction of the deposit (“vitelliruptive” stage). Irregular FAF signal (D), preserved elongation of photoreceptors (E, F) and fundus photograph of posterior pole in “vitelliruptive” stage (G). Figure S3-P17: FAF images of Best’s maculopathy patient (P17) (A), showing a lesion consisting of a circular hyperautofluorescent parafoveal circle with an annular hypofluorescent ring enclosing a second inner hyperautofluorescent ring and a hypoautofluorescent central spot (tip of the dome). The infrared images show hyperreflective foveal changes more visible in the LE (B bottom). The corresponding SD-OCT scans in both eyes (C) show subfoveal hyperreflective lipofuscin deposits at RPE level, surrounded by thin neurosensory retinal detachment, more prominent in the LE. The photoreceptor layer, partially intact in the RE and with slight disruption in the LE, is displaced on top of the lesion. In the LE tiny microcystic spaces subtending inner-layer retinal splitting can also be observed. Figure S4-P18: FAF (A, C) and OCT sca [file 12967_2019_2080_MOESM2_ESM.docx]

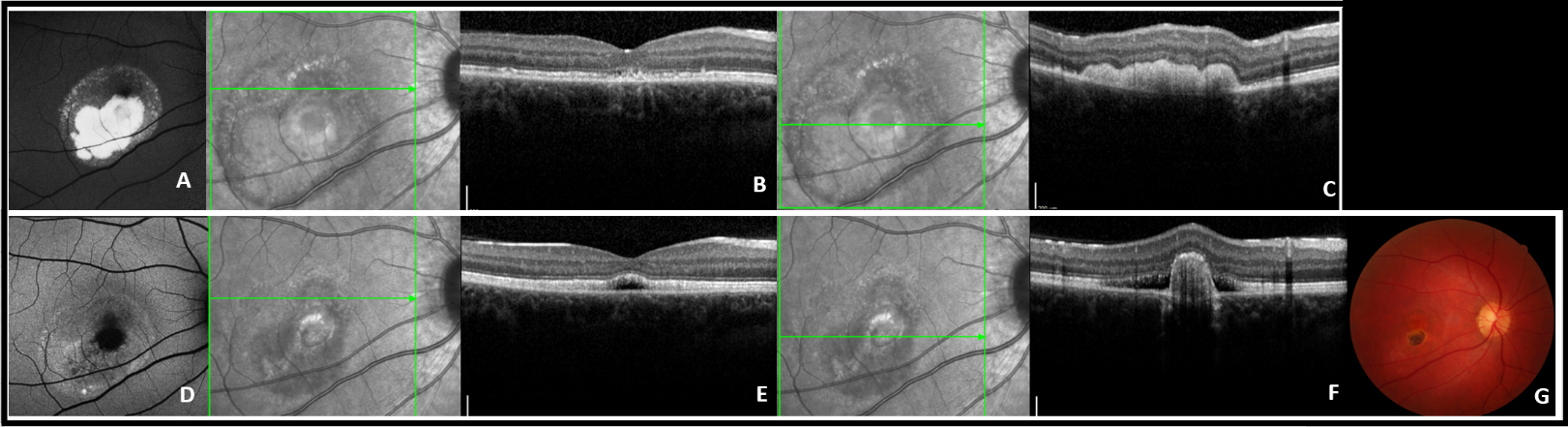


**Figure S1-P4:** Right eye five-year follow-up in a 22-year-old patient carrying a p.Val9Gly variant in *BEST1*. SD-OCT scans and corresponding FAF through the fovea and vitelliform deposit at baseline (A, B and C) and 5 years later (D, E and F). After 5 years the patient showed a “vitelliruptive” stage with irregular FAF signal (D), elongation of the outer segments under the fovea (E) and a thin neurosensory retinal detachment surrounding the lipofuscin deposit (E, F). Fundus photograph of the posterior pole at the last follow-up (G).


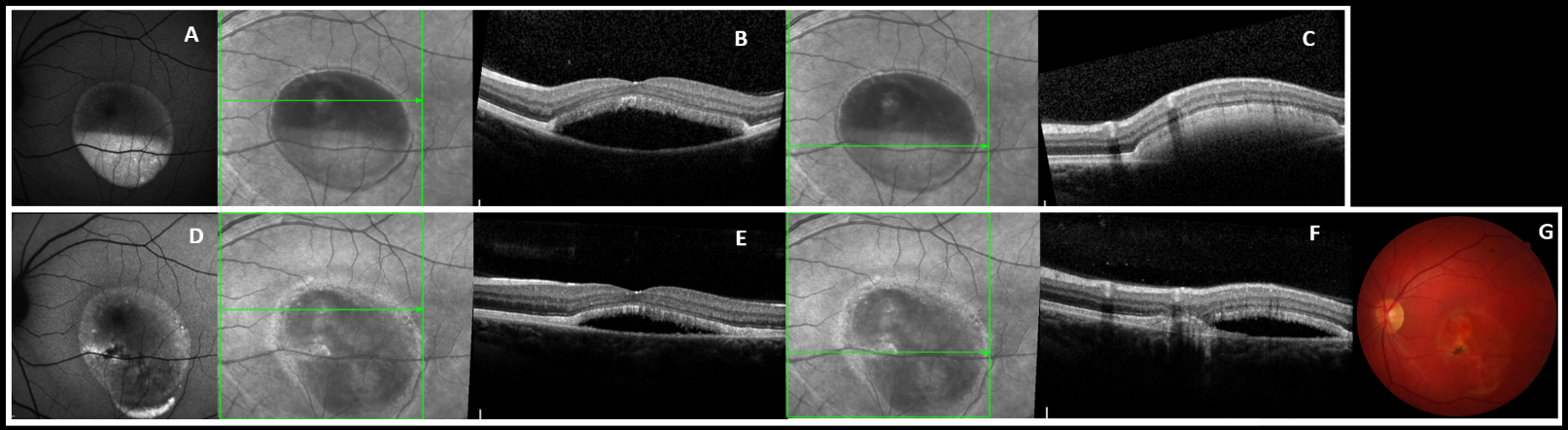


**Figure S2-P4:** Left eye five-year follow-up of the same patient. At baseline a “pseudohypopyon” stage is clearly visible in FAF (A) and OCT scans (B, C). Serous detachment of the neurosensory retina is visible in the upper macula with decreased FAF levels (Fig. 2B). In the bottom row of images, the yellow vitelliform material is evident by FAF. Five years later, there was reduction of the serous detachment and contraction of the deposit (“vitelliruptive” stage). Irregular FAF signal (D), preserved elongation of photoreceptors (E, F) and fundus photograph of posterior pole in “vitelliruptive” stage (G).


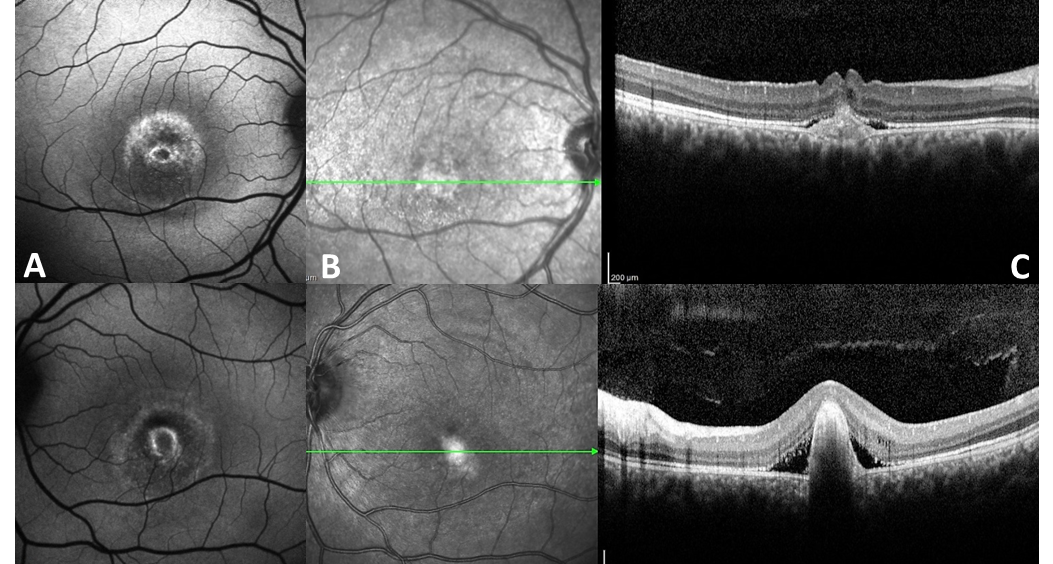


**Figure S3-P17:** FAF images of Best’s maculopathy patient (P17) (A), showing a lesion consisting of a circular hyperautofluorescent parafoveal circle with an annular hypofluorescent ring enclosing a second inner hyperautofluorescent ring and a hypoautofluorescent central spot (tip of the dome). The infrared images show hyperreflective foveal changes more visible in the LE (B bottom). The corresponding SD-OCT scans in both eyes (C) show subfoveal hyperreflective lipofuscin deposits at RPE level, surrounded by thin neurosensory retinal detachment, more prominent in the LE. The photoreceptor layer, partially intact in the RE and with slight disruption in the LE, is displaced on top of the lesion. In the LE tiny microcystic spaces subtending inner-layer retinal splitting can also be observed.


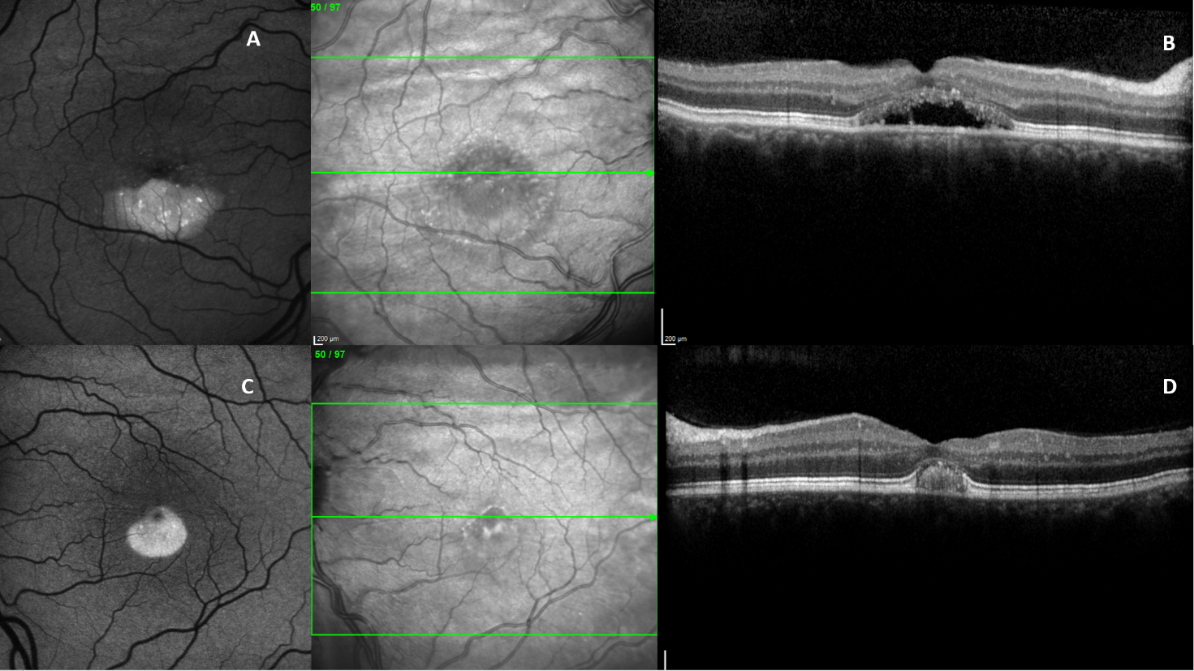


**Figure S4-P18:** FAF (A, C) and OCT scans (B, D) of a 46-year-old patient carrying a p.Asn179Asp variant in *BEST1*. Idiopathic choroidal folds and central accumulation of hyperautofluorescent lipofuscin is evident in both eyes. The right eye is in “pseudohypopyon” stage, as suggested by the neurosensory retinal detachment, while the left eye is still in *“*vitelliform” stage.


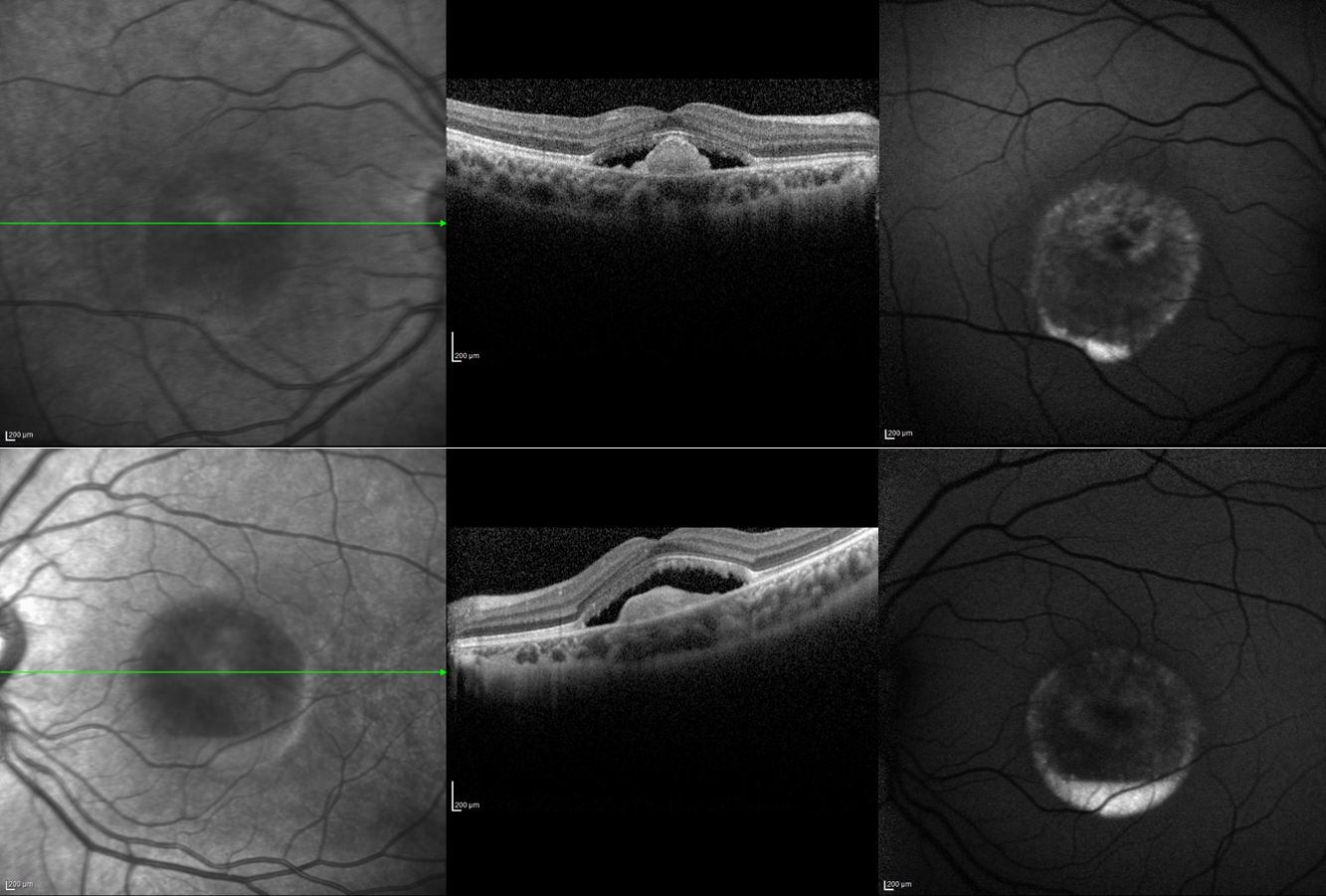


**Figure S5-P19:** FAF and OCT scans of a 13-year-old girl with a Trp182Arg variant in *BEST1*, showing bilateral vitelliform lesions at pseudohypopyon stage in both eyes. OCT scans show subfoveal hyperreflective lipofuscin deposits at RPE level, surrounded by thin neurosensory retinal detachment.


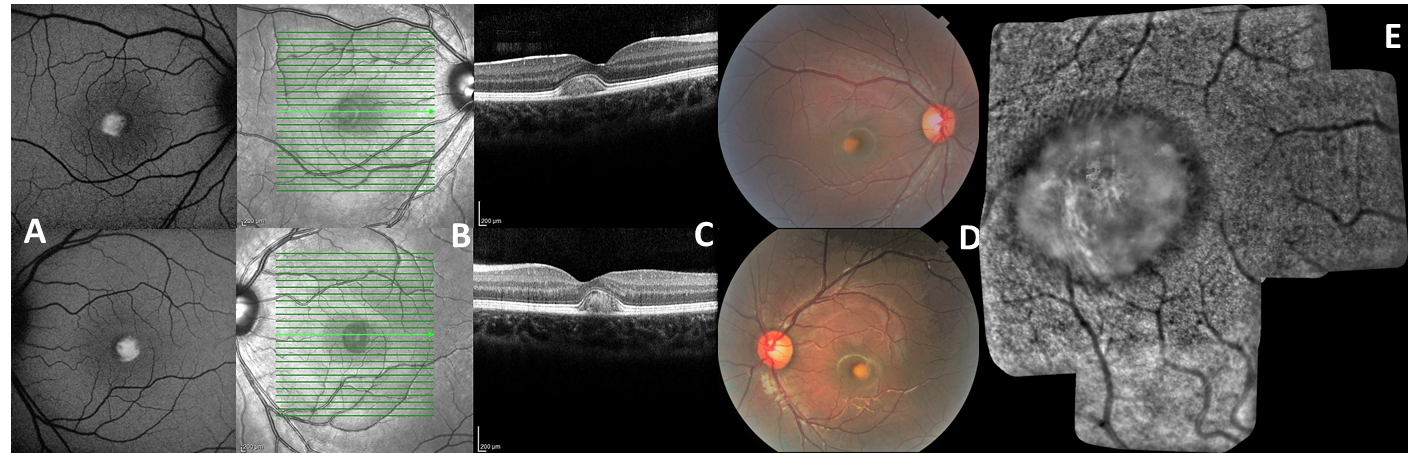


**Figure S6-P31:** Patient 31 (P31), a 9-year-old boy, showing fundus ophthalmoscope evidence of bilateral circular yellowish yolk-like lesions in the macular area (D). The lesions are symmetrically hyperautofluorescent with a nasal hypoautofluorescent sickle visible by FAF (A). At the same location in IR images, there is a circular area of hyporeflectivity matching the lipofuscin deposit at sub-RPE level with consequent reorganization of the IS-OS junction layer on top of the dome, as seen also in OCT scans (C). Preservation of the photoreceptor layer is also confirmed by adaptive optical images, showing cone mosaic structure in the RE (E), where the lesion appears elevated but with a normal arrangement of cones on top of it. In fact, intact bright cones can be seen across the central 6 degree eccentricity around the fovea, as well as a circular dark ring of shadow delineating the contour line of the lesion, where faint, still resolvable cones seem mechanically distorted.
